# What Types of Networks Do Professionals Build, and How Are They Affected by the Results of Network Evaluation?

**DOI:** 10.3389/fpubh.2021.758809

**Published:** 2021-11-23

**Authors:** Junji Haruta, Sho Tsugawa

**Affiliations:** ^1^Medical Education Center, School of Medicine, Keio University, Tokyo, Japan; ^2^Department of Primary Care and Medical Education, Faculty of Medicine, University of Tsukuba, Tsukuba, Japan; ^3^Division of Information Engineering, Faculty of Engineering, Information and Systems, University of Tsukuba, Tsukuba, Japan

**Keywords:** community medicine, home medical care, interprofessional relationships, mixed methods, social network analysis, content analysis

## Abstract

**Background:** We aimed to explore what kind of social networks characterizable as “consult/be consulted” are built among healthcare professionals in a community and the impact of providing the professionals with these findings.

**Methods:** We adopted mixed methods exploratory study using social network analysis (SNA) and content analysis. SNA can visualize social network structures such as relationships between individuals. The healthcare professionals were asked about the key persons they consulted and were consulted by concerning these healthcare issues: (1) daily work; (2) a person with acute back pain; (3) a garbage-filled house reported by a neighbor; (4) a person with dementia; and (5) a study meeting. We identified the key roles depending on the issues using SNA. After analysis, the analytical findings were shared with the participants. To explore their cognitive responses, an open-ended questionnaire was delivered and a content analysis was implemented.

**Results:** Of 54 healthcare professional participants, the data of 52 were available for analysis. The findings (in the respective order of the five topics above) were as follows: the number of nodes was 165, 95, 85, 82, and 68; clustering coefficient was 0.19, 0.03, 0.02, 0.11, and 0.23; assortativity was −0.043, −0.11, −0.23, −0.17, and −0.23; reciprocity was 0.35, 0.31, 0.39, 0.29, and 0.48. The top three centralities included nurses. Eighty-seven free comments were received, of which 39 were categorized as descriptive, 10 as analytical, and 38 as critical.

**Discussion:** The structure of “consult/be consulted” networks differed by topic. SNA is available to detect the healthcare resources network and it may have helped them to reflect on their own networks.

## Introduction

As the number of elderly people and complexity of healthcare issues increases, problems such as degenerative change, multimorbidity, social deprivation and psychological distress become ever more prominent. Healthcare professionals dealing with such issues, in turn, need to provide integrated care in an interprofessional manner (1). Achieving this requires that healthcare professionals across organizations share information about the issues of patients, users, families, and communities (2). Against this background, Japan has proposed to establish a community-based integrated care system (3). Due for implementation in 2025, this policy aims to provide a variety of home, community-based, and institutional services for every Japanese person age 65 years or older, based strictly on physical and mental status. Implementation requires local governments to establish a network of human relationships through which information can be shared across professions and organizations (4). However, evidence on the efficacy of social networking which professions are sharing information, in what network structure, and with what roles among healthcare professionals in a community is lacking.

One common means of visualizing such network structures is social network analysis (SNA), but this has been little used in the healthcare field (5). SNA focuses on the relationships between individuals as nodes and the link structure of an information-sharing clinical network (6), and can also be used to explore the roles of nodes connected by one or more interdependent relationships (7). Analysis of links between nodes representing individual healthcare professionals allows the use of SNA findings to suggest the function of network structures (8). Social networks connected by links among multiple healthcare professionals may represent an opportunity to achieve knowledge translation, change in professional behavior and improvement in patient outcomes (9, 10). In particular, the issues to be handled in a community-based integrated care system include not only medical and psychosocial complex problems, but also study meetings to solve them. One of the keys is a social network structure in which the appropriate healthcare professionals take the lead according to these issues. However, we do not always understand who the key role is for these complex problems. The use of SNA to clarify the network structures and roles of nodes of professionals in community networks therefore appears worthwhile.

A previous systematic review of SNA in healthcare fields identified six papers on the network structure of information sharing in organizations (11). Of these, however, only one study took place in a primary healthcare context (12); the other five were set in tertiary level facilities, including hospital units and specialist care facilities such as hemodialysis centers or nursing homes. They included the identification of communication patterns between nurses that correlated with safety and quality outcome measures (13); communication patterns between doctors and nurses in medication advice-seeking networks (14); analysis of the association between the infection prevention process and social networks in dialysis centers (15); evaluation of the impact of communication and use of technology related to skin care and pressure ulcers in nursing homes (16); and modeling coordination in hospital emergency departments (17). To date, however, few studies have reported the impact of feeding back such findings obtained using SNA to healthcare professionals. According to Moon (18), deliberate reflection has three meanings: (1) to make sense of what is being learned (meaningful learning), (2) to learn more from the process of drawing out what has been learned through meaningful learning, and (3) to learn by organizing the information and knowledge that already exists. Using this theoretical framework, to reflect deliberately a community-based integrated care system in Japan, it is necessary to visualize networking by healthcare professionals in response to local community issues and to evaluate the impact of providing the findings as feedback to healthcare professional participants involved in the next stage of the research.

We therefore used SNA to explore the type of “consult/be consulted” social networks constructed among healthcare professionals to address common issues in a community. We also aimed to conduct a qualitative study to evaluate the impact of providing these findings to healthcare professionals, and then to integrate the findings of these two studies in a mixed-methods study.

## Methods

### Study Design

We adopted a mixed methods exploratory design using social network analysis (SNA) and content analysis (19). SNA was performed to analyze communication patterns based on issues in a network (20). In this study, we used a tool called “name generator” to explore the individuals belonging to networks (21). This tool asks individual participants a series of questions and uses the responses to produce lists containing the names of persons forming the individual's network. The tool is then used to collect personal networks and analyze the assumptions and boundaries that characterize them, such as identifying significant persons among individual human resources (22). We adopted the “name generator” to allow analysis of the network structure and relationships of nodes, and to compare network structures based on the topics of individual healthcare professionals distinguishable from among other members of the same professional groups. Finally, the impact of providing this information to participants as feedback was evaluated using an open-ended, self-administered questionnaire, selected to minimize bias. The tool and the questionnaire were mainly developed by JH, with advice from the ST.

### Setting

For convenient sampling, we selected City X, in which a study meeting focusing on healthcare issues had been established several years previously. City X is a core city in a region with a population of 95,000 and four general hospitals (23), and we anticipated that it would exert little historical influence or vanity bias on the study. The study meetings for healthcare professionals had been held regularly every 1–3 months from 2017 (24). Participants were all healthcare professionals, and included care managers and nurses, as well as social workers, pharmacists, physicians, dentists, and welfare staff. Among these, care managers play a key role in the planning of home-visit care services provided under the Japanese Long-Term Care Insurance System, the long-term care insurance component of Japan's public insurance system. In this service, multiple types of healthcare professionals provide care at the patient's home, including visiting nurses, visiting pharmacists, and visiting physicians and dentists. As the direct caregiver of the client, home care workers visit patients' homes to assist with meals, toileting, and bathing while care workers assist residents in facilities. Meanwhile, medical institutions employ various medical professions such as physicians, nurses, pharmacists, social workers, dentists, among others. Finally, as gateways to medical contact, medical clerks are responsible for handling reception appointment requests, prescription requests, and enquiries. These members work in multiple medical and nursing care facilities in the local area, while care managers additionally coordinate services using long-term care insurance and share patient-related information with healthcare professionals across the organization.

### Participants

In this local community, study meetings are held every 1 or 2 months to help attendees solve challenges they are facing or learn about new topics. Attendees are mainly healthcare professionals including medical staff and welfare staff, and few administrative staff. To recruit participants, we informed previous study meetings attendees in person or by e-mail that the next study meeting hence would be held as part of the study. We also directly encouraged healthcare professionals who attended a care conference in September 2019 to take part in the study as a local accessibility-based convenience sample.

### Data Collection

We collected the data of healthcare professionals who attended the first study meeting, held in September 2019. The first author (JH) had been making weekly visits to this community since April 2019, but with a few exceptions, the study meeting was the first time he had met the participants. JH is a general practitioner and has received training in qualitative research as part of a PhD program. JH explained the purpose and content of the study to the participants and distributed the paper questionnaire. The participants were asked to provide their names, professions, and working places, and to identify key persons who they consulted with and were consulted by for healthcare issues in the following five topics: (1) daily work; (2) a person with acute back pain; (3) a garbage-filled house reported by a neighbor; (4) a person with dementia who increasingly wandered; and (5) a study meeting. These five topics were selected to identify social networks constructed among healthcare professionals to address common issues in the community, based on case studies of comprehensive care in Japan (25). To reduce response bias, JH informed participants that he would not see the raw data and that the findings would be analyzed after anonymization by the removal of individual names. All participants were informed about the study orally and with written information and provided written informed consent prior to being enrolled in the study. A research assistant conducted the data entry and ST (second author) analyzed the data.

After 5 months, JH provided the findings obtained up to that point as feedback to the healthcare professionals at the second study meeting, held in February 2020. Afterwards, the participants were asked to fill out a free-response questionnaire about the perceptions they gained from the findings using the question, “What did you notice or learn about the results of the SNA?”

### Constructing Networks

**Figure 1:**
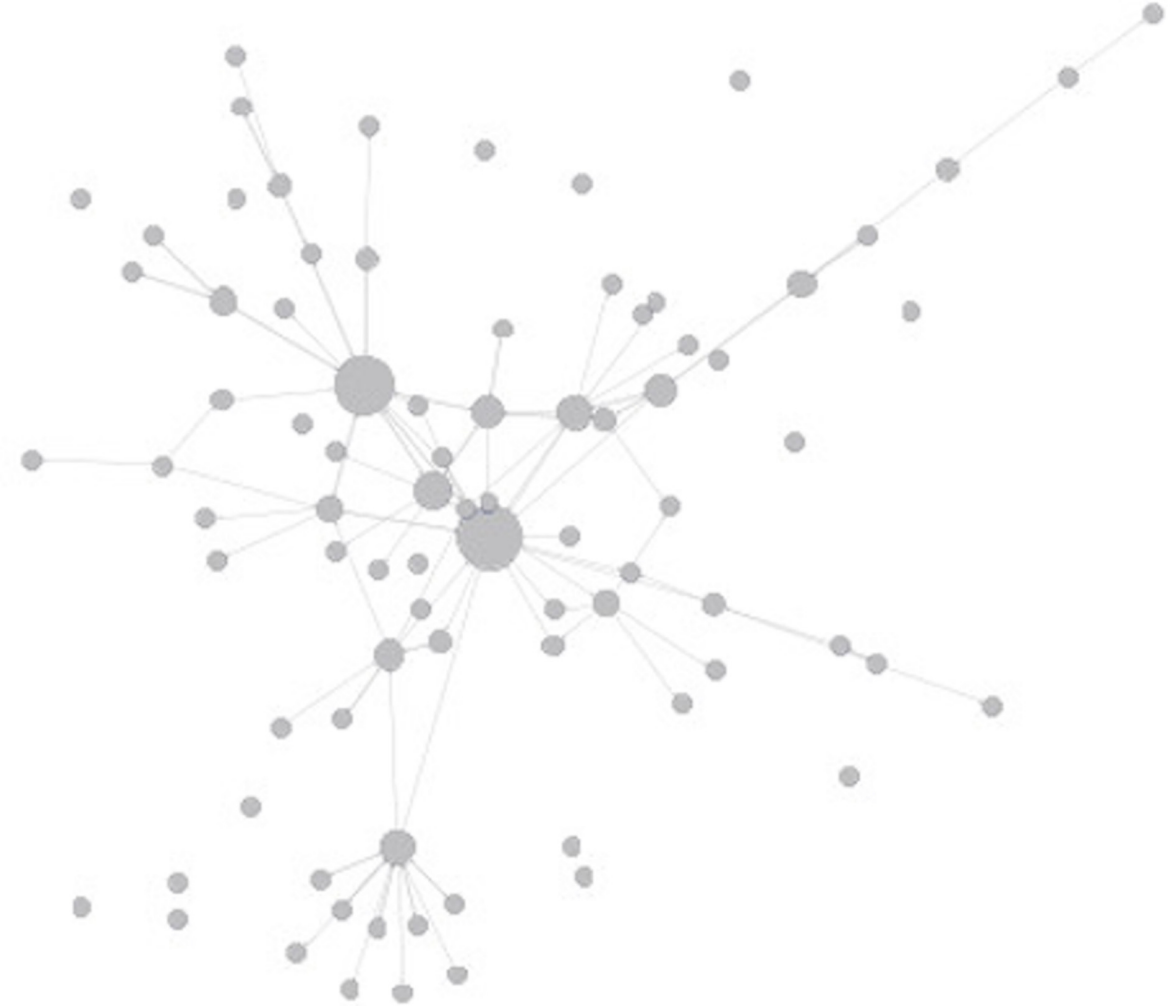
Visualization of social network patterns as a example.

Networks for each topic were constructed from the “consult/be consulted” key persons. The individual health professionals extracted for each topic were considered as nodes. The consult/be consulted relationships were considered as links. More specifically, for each topic, an unweighted directed graph *G* = *(V, E)* was constructed. Node *u* represents an individual healthcare professional, and a directed link *(u, v)* represents that individual healthcare professional *v* was consulted by *u*. As an example, a visualization of some of the social network patterns in this study is shown in Figure 1.

### Quantitative Phase; Social Network Analysis

ST is an expert in network analysis. SNA was conducted by ST in the five topics for nodes distinguished by their name, profession, and working place, and key persons who they consulted or who they were consulted by for any of the five healthcare issues. First, several measures of SNA were used to investigate the network structure for each healthcare issue. For each target network, the number of nodes, density (26), clustering coefficient (27), diameter (28), assortativity (29), reciprocity (30), and degree centralization, closeness centralization and betweenness centralization were determined (6). Density is the actual number of links in the network divided by the maximum possible number, as calculated using the software R igraph, and was determined to provide a more comprehensive description of the level of connectivity in the network (30). Density indicates that the larger the value, the more nodes are connected in the network. The clustering coefficient quantifies the abundance of connected triangles in a network and is useful in characterizing the clustering nature of a network (27). The high clustering coefficient means that a person who is connected to two other people has a high probability of being connected to the other person as triangular relationships. Diameter is the length of the longest and shortest paths in the network (28). That is, a network with a small diameter and an average distance can be considered “compact,” while a small number of nodes and a few long distances will have a large diameter. Assortativity is defined as the extent to which the nodes of a graph are linked–for example in “consult/be consulted” relationships–to other nodes which possess a similar number of connections (29). That is, high assortativity means that those with many connections associate with others with many connections. On the other hand, low assortativity means that well-connected individuals are connected to many individuals with few other connections. Reciprocity is an indicator of the proportion of bidirectional links in a network (30). That is, high reciprocity indicates the network has more bidirectional relationships.

Degree centralization, closeness centralization, and betweenness centralization, which are widely used in SNA, were obtained from the deviations of centrality measures of nodes: degree centrality is the bias in the number of links per node; closeness centralization is the deviation of the distance from all nodes; and betweenness centrality is the bias in the percentage of nodes that must be passed through to reach other nodes (6). In a network with high centralization, there are highly central nodes compared with other nodes, whereas in a network with low centralization, nodes have similar centrality.

Second, we investigated the key participant(s) in each topic by extracting the top-three nodes based on centrality measures in the network. For each of four centrality measures (indegree, outdegree, closeness, and betweenness), the nodes with the highest centrality for the five topics were extracted. Note that multiple nodes were extracted for each topic's network when four or more nodes had the top-three centralities. We calculated the indegree, outdegree, centrality, closeness centrality, and betweenness centrality measures for each node in each network and identified the top three nodes of professionals according to their ranking.

### Qualitative Phase; Content Analysis

Free text responses to the free-response questionnaire were entered into an Excel file by the research assistant. Excel data from which the names were removed were analyzed by JH using inductive content analysis (31). Coding and theme extraction were labeled according to the content of the comments. JH read all responses and each comment, identified the meaning, and extracted individual potential codes. In further structuring of the data, the authors articulated a subtheme in the form of a question about how the comments were interpreted from the data according to the general critical thinking classification (32). Based on the classification, the themes were categorized as descriptive, analytical, and critical in order to clarify their cognitive impact on the participants. A comment that explained a theme descriptively according to the SNA's findings but lacked integration with interpretation was categorized as descriptive. If the comment was integrated with an analytic interpretation, it was categorized as analytical. A report that further reflected on one's own ideas about the findings was categorized as reflective. JH independently assessed the levels of classification; ST determined whether or not the interpretation of the data was reasonable; and finally, JH and SH agreed on three categories of critical thinking classification which emerged from the free comments. This study was approved by the Ethics Committee in author's University.

## Results

The individual healthcare professionals who attended the first and second study meetings in City X were characterized as follows (Table 1).

**Table 1:** Characteristics of individual healthcare professionals.

	**First study meeting**		**Second study meeting**	
**Healthcare professional**	**Number**	**%**	**Number**	**%**
Care manager	13	24.1	7	20.0
Nurse	11	20.4	6	17.1
Pharmacist	11	20.4	7	20.0
Social worker	8	14.8	5	14.3
Physician and dentist	3	5.6	5	14.3
Administration staff	3	5.6	0	0
Facility manager	2	3.7	1	2.9
Public health nurse	2	3.7	1	2.9
Home care worker	1	1.9	0	0
Medical clerk	0	0	1	2.9
Unknown	0	0	2	5.7
Total	54	100	35	100

### Quantitative Findings

Of the 54 participants in the first study meeting, the data of 52 were available for analysis.

Findings in the respective order of (1) daily work; (2) a person with acute back pain; (3) a garbage-filled house reported by a neighbor; (4) a person with dementia who increasingly wandered; and (5) a study meeting were presented as follows: the number of nodes was 165, 95, 85, 82, and 68; diameter was 4.6, 3.8, 3.1, 3.3, and 3.6; cluster coefficient was 0.19, 0.03, 0.02, 0.11, and 0.23; density was 0.0090, 0.013, 0.016, 0.014, and 0.027; assortativity was −0.043, −0.11, −0.23, −0.17, and −0.23; reciprocity was 0.35, 0.31, 0.39, 0.29, and 0.48; degree centrality was 0.12, 0.23, 0.28, 0.16, and 0.30; closeness centrality was 0.31, 0.34, 0.39, 0.43, and 0.41; and betweenness centrality was 0.39, 0.18, 0.15, 0.22, and 0.52 (Table 2). The diameter was ordered by daily work, acute back pain, study meeting, dementia, and garbage house. Clustering coefficient and assortativity are relatively low in all networks. That is, there was few triangular relations and bias in the number of connections. The social network of study meetings tended to be high in reciprocity, degree centralization, closeness centralization, and betweenness centralization.

**Table 2:** Overall network structure.

	**Daily work**	**Acute back pain**	**Garbage-Filled house**	**Dementia**	**Study meeting**
Number of nodes	165	95	82	85	68
Diameter	10	9	6	6	8
Clustering coefficient	0.19	0.03	0.02	0.11	0.23
Density	0.009	0.013	0.016	0.014	0.027
Assortativity	−0.043	−0.11	−0.23	−0.17	−0.23
Reciprocity	0.36	0.31	0.39	0.29	0.48
Degree centralization	0.12	0.23	0.28	0.16	0.3
Closeness centralization	0.31	0.34	0.39	0.43	0.41
Betweenness centralization	0.39	0.18	0.15	0.22	0.52

Among topics, it became clear that the core of the network was mainly nurses and social workers for daily work, acute back pain, garbage-filled house, and dementia. The top one, other than study meeting and outdegree in daily work, was nurse. In the network of daily work, the top 1 in the outdegree and the top 2 in betweenness and closeness centralization were social workers. Physicians and nurses played key roles in the network of the study meetings. In the network of study meeting, nurses remained the top 1, but the top 2 for betweenness and closeness centralization and the top 3 for indegree were physicians (Table 3).

**Table 3:** Professionals with the highest three nodes for network centrality.

		**Top 1**	**Top 2**	**Top 3**
Daily work	Indegree	Nurse	Nurse	Social worker
	Outdegree	Social worker	Nurse	Pharmacist
	Betweenness	Nurse	Social worker	Nurse
	Closeness	Nurse	Social worker	Physician
Acute back pain	Indegree	Nurse	Nurse	Pharmacist
	Outdegree	Nurse	Nurse	Pharmacist
	Betweenness	Nurse	Nurse	Social worker
	Closeness	Nurse	Nurse	Social worker
Garbage-Filled house	Indegree	Nurse	Nurse	Social worker
	Outdegree	Nurse	Nurse	Social worker
	Betweenness	Nurse	Nurse	Care manager
	Closeness	Nurse	Nurse	Pharmacist
Dementia	Indegree	Nurse	Nurse	Care manager
	Outdegree	Nurse	Nurse	Nurse
	Betweenness	Nurse	Nurse	Social worker
	Closeness	Nurse	Nurse	Nurse
Study meeting	Indegree	Nurse	Nurse	Physician
	Outdegree	Nurse	Physician	Nurse
	Betweenness	Nurse	Physician	Nurse
	Closeness	Nurse	Physician	Pharmacist

### Qualitative Findings

We received 87 free comments, of which 39 were categorized by theme as descriptive, 10 as analytical, and 43 as critical. Descriptive comments provide introductory and background/contextual information. These texts were mostly labeled with the code of awareness of the current situation that addressed questions such as “what is this about?” and/or “how did this occur?” Analytical comments explored relationships of ideas or parts of something. These texts were labeled with a code to represent the exploratory relationships of their ideas and provide a possible situation based on findings for current social networks constructed among healthcare professionals, such as “why did this occur?” and/or “what if there was a problem?” and/or “why not something else?” Critical comments characteristically evaluated the context, outlined the meaning and value, and explored the outcome. These texts were labeled with the code, which reflected the findings from many different angles such as “what does mean? “and/or “what can be learnt for applying in the future?” and/or “what are the implication?” and/or “what did I notice?” (Table 4).

**Table 4:** Content analysis of free comments.

**Examples of comments**	**Code**	**Subtheme**	**Theme**	**Number (total 87)**
“Many medical professionals (not care managers) are at the center of the network”	Awareness of the current situation that had hitherto been latent	What is this about?/How did this occur?	Descriptive	39
“There are many people working in different professions, but they are not connected through networks because there is no opportunity for them to engage with others on some topics”	Interpreting the mechanisms of the current situation as manifested	Why did this occur?/What if there was a problem?	Analytical	2
“It became apparent that they often did not know what advice to ask for because they did not understand the work of the professionals”	Awareness of the emerging findings and associated challenges	Why not something else?		3
“I think there are many dementia patients living in the community, but I was surprised to see that they are all individual and not connected to each other”	Awareness of interprofessional collaboration to meet local needs	What if there was a problem?		5
“(The fact that it seems to function smoothly even when professionals are not connected) I found out that the local community is very fortunate to have many informal people such as volunteers and local residents”	View something from many different angles	What does this mean?	Critical	3
“There is still a lot of room for growth in this local community. I wanted to increase the number of lines (hand-holding) one by one”		What can be learnt for future application?		4
“There are many times when I find myself struggling with difficult cases on my own. In such cases, I would like to consult with a specialist to solve the problem”	Clarifying outlines implications and solutions	What are the implications?		8
“I thought that nurses who are involved in homecare are often consulted. I think it will be a wonderful local community if welfare staff, doctors, and pharmacists could work together more.” “I felt that it is important to know what kind of work many professions are doing, while building relationships through study meetings and connecting with them”	Making a judgment on the quality of something	What can be learnt for future application?		10
“I reflected that pharmacists, in particular, may have ended up in the pharmacy.” “I guess that I couldn't think of anyone to talk to about a problem that was beyond my own imagination”	Self-reflection guided by revealed findings	What did I notice?		13

## Discussion

Using SNA, we clarified the network structures of “consult/be consulted” relationships among healthcare professionals, and identified the key roles which differed depending on the specific healthcare issue. When the findings were fed back to the healthcare professionals, it became clear that the healthcare professionals mainly responded descriptively and critically, whereas a relatively few participants responded analytically.

The clustering coefficients were low in every network in the community. This could be inferred from the fact that there were few relationships in which three or more people consulted each other. These findings suggest that even if consultation networks are present among healthcare professionals, individual healthcare professionals may tend to communicate with each other in limited and closed relationships. In a community-based integrated care system that needs to deal with various complex issues such as medical welfare and psychosocial issues, this network structure may have its limitations. Compare to the previous studies (33, 34), the combination of a low clustering coefficient and a diameter of 3.1–4.6 indicated that serendipitous or chance interactions among healthcare professionals were unlikely.

Among these networks, those for study meetings and daily work had relatively high clustering coefficients, suggesting that there are relatively many relationships in which groups of three or more people consult each other. In the other hand, for specific problems such as acute back pain, a garbage-filled house, and dementia, the relationship was more likely to be a one-on-one consultation relationship. Additionally, the networks for acute back pain and a garbage-filled house included weaker betweenness centralization than those for study meetings and daily work. That is, the lower clustering coefficient and weaker betweenness networks had few key players who played a role in connecting the fragmented network. Although the network for dementia was similar to those for acute back pain and garbage-filled house with regard to density and assortativity, its clustering coefficient and betweenness centralization were relatively smaller and degree centralization was relatively larger than that for daily work. That is, the characteristics of a network structure for dementia may lie somewhere between acute back pain and daily work. Since the number of consultations for dementia in Japan is increasing in the community in line with the super-aging of society (35), healthcare professionals may tend to consult more specific key persons than they do for acute back pain and a garbage-filled house.

From a micro perspective, the professionals with the highest betweenness centralization were nurses in every topic. Nurses have also been reported to be important in the role of network centrality (36, 37). Although subject to local characteristics, community nurses play an important role in connecting communities outside of hospitals, and may be required to play a generic role (38). This might simply reflect the characteristics of the issues that each profession is likely to face. As a whole, the findings of the weakness of overall connections may reflect the short history of network building among healthcare professionals in this local community. However, even if the connections of nodes are weak in consultation networks for dementia, such as for pharmacists, these nodes may have opportunities to identify some specific nurses as playing key roles in a community, and the network for these issues may expand as time passes (39). Other studies using SNA have demonstrated that central, influential, and well-connected nodes are not only easily identifiable but also clarify the “hidden” key players (40). Based on this, even if other staff have few opportunities to engage with such healthcare issues in a community, the use of SNA findings may help them understand the roles of other professionals, and to acknowledge and support their own roles (41).

The findings of this study had a critical impact on the participating healthcare professionals, and lead to descriptive and critical comments for next actions from them. In contrast, the analytical impact was low. This may be related to or reflect the weakness reported in the analytical perspective of healthcare professionals (42). Nevertheless, sharing of the findings of the SNA with healthcare professionals may have provided them with an opportunity to reflect and promoted innovative thinking. It has been reported that appropriate feedback to healthcare professionals can be an opportunity to promote behavioral change (43). In addition, healthcare issues exemplified by the topics of acute back pain, a garbage-filled house and dementia may occur not only in Japan but also in other countries. Analysis of SNA in networks for specific topics in a community will likely provide an opportunity to reflect on community information sharing networks, such as the proposed community-based integrated care system in Japan. Accordingly, healthcare professionals should utilize SNA to evaluate social capital among healthcare professionals in communities facing complex issues.

One limitation of this study is its use of convenience sampling in City X. Although social networks should be considered to encompass the design of the entire network, healthcare participants in the study were limited to those who participated in the study meetings, rather than all healthcare professionals in the community. In addition, we cannot deny the possibility that the results reflect the specific characteristics of this local community. However, it is worth noting that the network structure of the “consult/be consulted” relationship differed depending on the five common topics in this local community. Our present SNA-based method needs verification in other communities. With regard to implementation, the fact that the participants were given clear examples of how to “consult/be consulted” on specific healthcare issues was easily understood by the participants, which might have in turn enhanced their cognitive impact, such as with regard to critical comments. Thus, the methodology of this study, which used questionnaires to explore human relationships in a community and provide the findings as feedback, can provide evidence for the establishing, maintaining, and rebuilding of community information sharing networks. We believe that the methodology of this study will be worthwhile for many developed countries facing aging societies, and for Asian countries with a similar cultural background to Japan.

## Conclusion

We explored the network structure of an information sharing network in a community as “consult/be consulted” relationships using SNA. Differences in social network structure were clarified using specific healthcare issues, and the key role of nurses in the networks emerged. The impact of feeding back the findings to healthcare professionals may have helped them to reflect on their own networks, and to solve issues with them.

## Data Availability Statement

The datasets presented in this article are not readily available because we did not receive informed consent concerning data sharing from the participants. Requests to access the datasets should be directed to Junji Haruta, junharujp@keio.jp.

## Ethics Statement

The studies involving human participants were reviewed and approved by Ethics Committee of the Faculty of Medicine, University of Tsukuba (No. 1429). The patients/participants provided their written informed consent to participate in this study.

## Author Contributions

JH and ST were involved in the conception and design of this study and analyzed the data. JH collected data and mainly wrote the paper. ST revised it critically for important intellectual content and both of them approved the paper.

## Funding

This work was supported by JSPS KAKENHI Grant-in-Aid for Young Scientists (B) Grant No. JP19K19377.

## Conflict of Interest

The authors declare that the research was conducted in the absence of any commercial or financial relationships that could be construed as a potential conflict of interest.

## Publisher's Note

All claims expressed in this article are solely those of the authors and do not necessarily represent those of their affiliated organizations, or those of the publisher, the editors and the reviewers. Any product that may be evaluated in this article, or claim that may be made by its manufacturer, is not guaranteed or endorsed by the publisher.
